# MRI Detection and Therapeutic Enhancement of Ferumoxytol Internalization in Glioblastoma Cells

**DOI:** 10.3390/nano14020189

**Published:** 2024-01-13

**Authors:** Michael S. Petronek, Nahom Teferi, Chu-Yu Lee, Vincent A. Magnotta, Bryan G. Allen

**Affiliations:** 1 Department of Radiation Oncology, University of Iowa, Iowa City, IA 52242, USA; 2 Department of Neurosurgery, University of Iowa, Iowa City, IA 52242, USA; nahom-teferi@uiowa.edu; 3 Department of Radiology, University of Iowa, Iowa City, IA 52242, USAvincent-magnotta@uiowa.edu (V.A.M.)

**Keywords:** ferumoxytol, glioblastoma therapy, glioblastoma imaging, pha

## Abstract

Recently, the FDA-approved iron oxide nanoparticle, ferumoxytol, has been found to enhance the efficacy of pharmacological ascorbate (AscH^−^) in treating glioblastoma, as AscH^−^ reduces the Fe^3+^ sites in the nanoparticle core. Given the iron oxidation state specificity of T2* relaxation mapping, this study aims to investigate the ability of T2* relaxation to monitor the reduction of ferumoxytol by AscH^−^ with respect to its in vitro therapeutic enhancement. This study employed an in vitro glioblastoma MRI model system to investigate the chemical interaction of ferumoxytol with T_2_* mapping. Lipofectamine was utilized to facilitate ferumoxytol internalization and assess intracellular versus extracellular chemistry. In vitro T_2_* mapping successfully detected an AscH^−^-mediated reduction of ferumoxytol (25.6 ms versus 2.8 ms for FMX alone). The T_2_* relaxation technique identified the release of Fe^2+^ from ferumoxytol by AscH^−^ in glioblastoma cells. However, the high iron content of ferumoxytol limited T2* ability to differentiate between the external and internal reduction of ferumoxytol by AscH^−^ (ΔT_2_* = +839% for external FMX and +1112% for internal FMX reduction). Notably, the internalization of ferumoxytol significantly enhances its ability to promote AscH^−^ toxicity (dose enhancement ratio for extracellular FMX = 1.16 versus 1.54 for intracellular FMX). These data provide valuable insights into the MR-based nanotheranostic application of ferumoxytol and AscH^−^ therapy for glioblastoma management. Future developmental efforts, such as FMX surface modifications, may be warranted to enhance this approach further.

## 1. Introduction

Ferumoxytol (Feraheme^®^, FMX) is a clinically available, superparamagnetic iron oxide nanoparticle approved for treating iron deficiency anemia in patients with chronic kidney disease [1,2,3,4]. FMX can generate T1-contrast enhancement in tumor tissue in glioma imaging due to its ferromagnetic properties. FMX is a superparamagnetic iron oxide nanoparticle (SPION) with a Fe_3_O_4_ core that is about 30 nm in size, has a neutral charge, and resides within a carboxylated polymer coating [5]. Many units of the Fe_3_O_4_ core exist in one nanoparticle yielding a wide range of molecular weights with an average of about 730 kDa [6]. Because of the large iron content of one molecule of FMX (1 molecule has ≈ 5900 iron atoms or 1 nM FMX ≈ 5.9 µM iron), it can function as a T_1_/T_2_* MRI contrast agent [7,8]. Ferumoxytol’s iron content and ferromagnetic properties also allow its use as a T_2_*-contrast agent because T_2_* relaxation times are largely influenced by paramagnetic and ferromagnetic materials (e.g., iron). FMX’s superparamagnetic properties alter T_2_* relaxation times [9,10]. FMX is an attractive MR contrast agent because it has a significantly longer intravascular half-life (*t*_1/2_ ≈ 14–21 h) than gadolinium-based compounds (*t*_1/2_ ≈ 1 h) [7,11].

Beyond its utility as an MRI contrast agent, FMX has shown potential as an anti-cancer therapy [12,13]. The anti-cancer mechanism of FMX has been suggested to be due to its redox activity. It has previously been shown that the Fe_3_O_4_ core can be oxidized by ionizing radiation, showing that FMX can serve as a reserve of redox-active iron [14]. FMX also reacts with H_2_O_2_ stimulating the release of iron from the nanoparticle. Thus, FMX may undergo redox reactions with a wide array of species. Ascorbate (AscH^−^) is a one-electron reductant that can readily reduce some complexes of ferric (Fe^3+^) to ferrous (Fe^2+^) iron [15]. AscH^−^ can reduce and release Fe^2+^ from ferritin, a Fe^3+^-containing biological macromolecule that is the primary mechanism for intracellular iron storage [16,17,18]. Recently, it has been reported that AscH^−^ catalyzes the decomposition of the FMX Fe_3_O_4_ core [19]. The chemical interaction between FMX and AscH^−^ can be characterized by a significant reduction in FMX size (≈66% reduction in 24 h), a release of redox-active Fe^2+^ that follows Michaelis–Menton kinetics, and a significant increase in H_2_O_2_ generation. The decomposition of FMX by AscH^−^ was reported to enhance glioblastoma cell killing and importantly, the enhanced toxicity of FMX and AscH^−^ was glioblastoma specific, as no significant in vitro toxicity was observed in normal human astrocytes [19]. Thus, the chemical pairing of FMX and AscH^−^ represents a novel therapeutic strategy. However, the utility of FMX as an MRI contrast agent suggests that FMX and AscH^−^ may have nanotheranostic potential.

T_2_* relaxation mapping is a quantitative MRI technique used primarily to indicate total iron content [20]. This technique is widely applicable clinically for cardiac and hepatic iron overload [21,22,23,24,25,26]. Recent studies have shown that beyond total iron content, T_2_* can provide information on the oxidation state of iron, specificity differentiating between Fe^3+^ and Fe^2+^ [27,28]. This effect is theorized to result from proton– electron dipole–dipole interactions associated with the number of unpaired electrons (i.e., electron spin magnetic moment) of transition metals [29]. Moreover, a recent phase 2 clinical trial testing AscH^−^ therapy in combination with radiation and temozolomide showed that patients with short T_2_* relaxation times (i.e., high iron content) had significantly greater therapy responses [30]. Because T_2_* relaxation appears to be largely dependent on the paramagnetic properties of metals and can detect alterations in these electronic spin properties (e.g., reduction/oxidation of iron), T_2_* mapping may serve as a useful tool in the evaluation of FMX redox chemistry. Therefore, changes in T_2_* relaxation may be reflective of the disruption of the FMX Fe_3_O_4_ core by AscH^−^. This study aims to provide detailed proof-of-concept insights into the ability of T_2_* mapping to evaluate Fe_3_O_4_ disruptions by AscH^−^ with respect to the in vitro toxicity of extracellular and intracellular FMX.

## 2. Materials and Methods

### 2.1. Cell Culture

Commercially available and validated U87, U251, and U118 glioblastoma cells were cultured in DMEM-F12 media (15% FBS, 1% penicillin-strep, 1% Na-pyruvate, 1.5% HEPES, 0.1% insulin, and 0.02% fibroblast growth factor) and grown to 70–80% confluence at 21% O_2_ before experimentation. Cells were treated for 1 h with AscH^−^ (20 pmol cell^−1^; ≈8–10 mM), diluted from a 1 M stock of AscH^−^ in H_2_O with pH = 7, in complete cell culture media without FBS or Na-pyruvate to prevent scavenging of H_2_O_2_. Cells were treated with 100 µM deferoxamine mesylate salt (DFO; Sigma, St. Louis, MO, USA; D9533) from a 110 mM stock in H_2_O. FMX was used from the commercially available Feraheme^®^ (30 mg mL^−1^ stock in saline).

### 2.2. In Vitro MRI Studies

Glioblastoma cells were treated with 20 pmol cell^−1^ AscH^−^ for 1 h with 20 µg mL^−1^ FMX or pre-incubated for 24 h with 20 µg mL^−1^ FMX-L prior to the 1 h AscH^−1^ treatment. Following treatment, cells were trypsinized, re-suspended in sterile PBS, and transferred to PCR wells embedded in a 1% agarose gel phantom. Cells were allowed to collect at the bottom of the PCR well to form a pellet to be imaged. Cell pellets were then imaged on a 7T GE MR901 small animal scanner, a part of the small animal imaging core at the University of Iowa. T_2_* weighted images were collected using a gradient-echo sequence (TR = 10 ms, TE = 2.2, 8.2, 14.2, and 20.2 ms, matrix = 256 × 256, FOV = 25 × 20 mm, 2 signal averages). A B_0_ shimming routine was performed to limit the effect of macroscopic field inhomogeneities. T_2_* maps were generated using a combination of 4 echo times collected and fitting each voxel to a mono-exponential curve using in-house Python code. Images were imported to 3D Slicer software (V5.0.3) where regions of interest (ROIs) were delineated as a 1 mm diameter cylinder in the center of the tube and mean T_2_* values were calculated using the label statistics tool within 3D Slicer [31].

### 2.3. FMX Internalization with Lipofectamine

Lipofectamine FMX (FMX-L) was generated using the commercially available lipofectamine 3000 reagents (Thermofisher Scientific, Waltham, MA, USA; L3000015). Functionalization was completed by diluting FMX at 1:16 in 1% FBS containing DMEM-F12 media (1 mL) with 10 µL P3000 reagent, vortexing vigorously for 5 s, and then diluting the FMX/P3000 stock at 1:1 with lipofectamine 3000. The samples were incubated at room temperature for 15 min prior to utilization. FMX-L was generated new for every experiment. The cells were then treated with FMX-L for 24 h in 1% FBS containing DMEM-F12 medium. The cells were washed with 1X D-PBS prior to additional studies to remove extracellular FMX.

### 2.4. Quantitation of Intracellular Iron

Intracellular iron concentrations were validated colorimetrically following a 24 h treatment with either 20 µg mL^−1^ FMX or FMX-L using a ferrozine-based assay [32,33]. Following treatment, cells were washed with sterile PBS, trypsinized, and centrifuged at 1200 rpm for 5 min. The cell pellets were resuspended in 1X RIPA buffer (Sigma-Aldrich, St. Louis, MO, USA; R0278) and sonicated 3 × 10 s to lyse the cells. Cell lysis solution was then diluted 1:1 in 2.5 M glacial acetic acid pH = 4.5 with 5 mM ferrozine and 10 mM AscH^−^. The sample and buffer mixture were centrifuged at maximum speed (14,000× *g*) for 10 min to remove protein aggregates. The supernatant (200 µL) was placed in a 96-well dish [33]. Ultraviolet-visible light (UV-Vis) spectroscopic measurements were performed using a 96-well plate reader. Fe^2+^ (ferrozine)_3_ complex formation was monitored by analyzing absorbance at 562 nm. Fe^2+^ concentrations were determined using Beer’s Law for absorbance at 562 nm (ε_562_ = 27,900 M^−1^ cm^−1^) with a path length, of L = 0.55 cm (200 µL sample).

### 2.5. Cellular Iron Staining

To visualize the iron deposition following FMX treatment, cells were stained using a Prussian Blue technique using an iron staining kit (Abcam, Cambridge, U.K.; ab150674) using the manufacturer’s protocol. Following treatment, cells were washed with 1X D-PBS and fixed with formalin for 5 min. The cells were then washed with distilled H_2_O and incubated for 15 min with a 1:1 mixture of potassium ferrocyanide and 2% hydrochloric acid. After staining, cells were washed with distilled H_2_O and stained for 5 min with a nuclear-fast red counterstain. Finally, cells were washed with distilled H_2_O and allowed to dry. The cells were then imaged using a phase contrast microscope with a 40× objective lens.

### 2.6. Electron Paramagnetic Resonance Evaluation of FMX Concentrations in Cell Culture Media

The FMX concentrations were determined by measuring the peak-to-peak signal intensity of the EPR spectrum of the low-spin Fe_3_O_4_ complex at *g* ≈ 2 as previously described [14]. Using a Bruker EMX spectrometer, the following scan parameters were used to collect spectra: center field = 3508.97 G, sweep width = 2000 G, frequency = 9.85 GHz, power attenuation = 18 dB, modulation frequency = 100 kHz, modulation amplitude = 0.7 G, with spectra being generated from a signal average of 2 scans with 2048 resolution. U87 cells were incubated for 24 h with 20 µg mL^−1^ FMX or FMX-L.

## 3. Results

### 3.1. In Vitro Oxidation State Specificity of T_2_* Mapping

**Figure 1:**
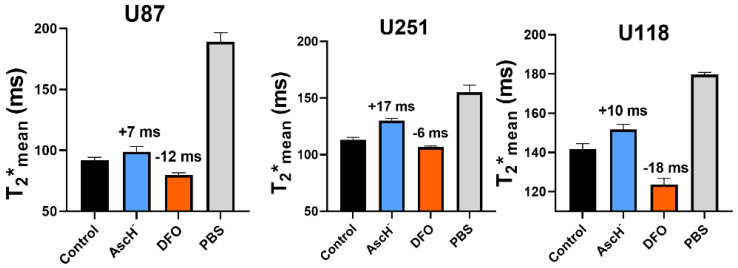
Pharmacological perturbations of intracellular iron can be detected in GBM cells using T_2_* mapping. Quantification of in vitro T_2_* maps of human GBM (U87, U251, U118) cells treated with P-AscH^−^ (20 pmol cell^-1^; range: 6–8 mM, 1 h) or DFO (200 µM, 24 h). Phosphate-buffered saline without cells was used as a positive control. Values represent the average magnitude of deflection in T_2_* relaxation from control (n = 3).

Before evaluating if T_2_* mapping can detect FMX and AscH^−^ chemistry, the in vitro oxidation state specificity of T_2_* mapping was tested using a previously established MRI phantom model system [29]. It was observed that AscH^−^ increased T_2_* relaxation times in U87, U251, and U118 GBM cell lines by 7 ms, 17 ms, and 10 ms, respectively (Figure 1). This is consistent with the previously observed increase in T_2_* relaxation times following a pharmacological ascorbate infusion in GBM subjects [27]. Moreover, the iron chelator deferoxamine (DFO) causes a decrease (U87 = −12 ms, U251 = −6 ms, and U118 = −18 ms) in T_2_* relaxation times indicative of a paramagnetic shift as a result of ferrioxamine (DFO-Fe^3+^) complex formation. This is consistent with the ability of DFO to bind and maintain Fe in the +3 oxidation state (Fe^3+^) [34]. Thus, T_2_* mapping can detect iron oxidation state changes associated with the oxidation when complexed by DFO or internally reduced by AscH^−^.

### 3.2. Lipofectamine Enhances FMX Internalization

**Figure 2:**
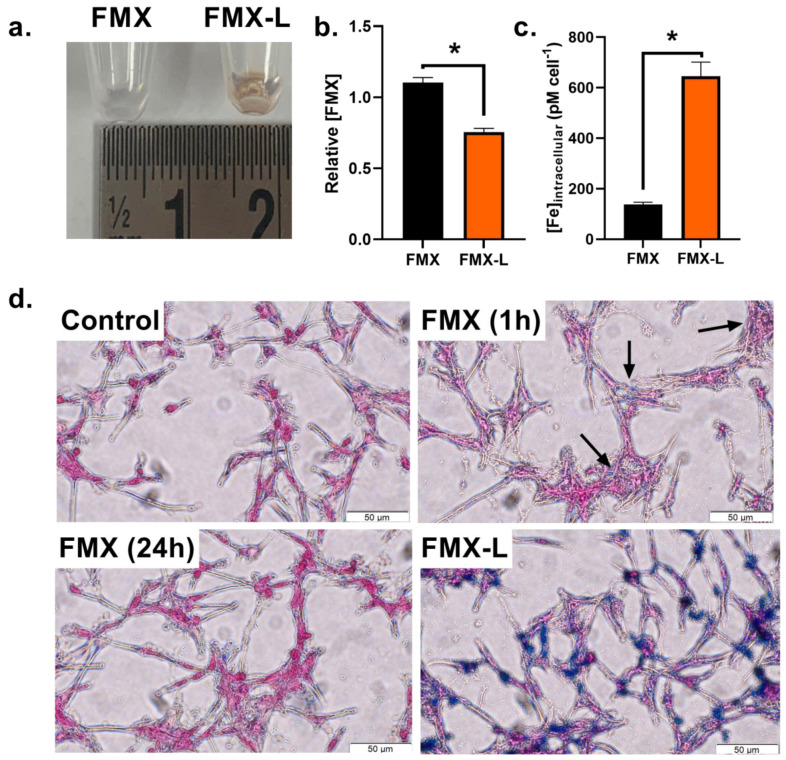
T_2_* mapping detects FMX internalization and reduction in vitro. (**a**) Cells were treated for 24 h followed by PBS washing and trypsinization. The large increase in intracellular iron content of FMX-L becomes apparent due to the reddish hue of the cell pellet. (**b**) Relative [FMX] concentrations in cell culture media following 24 h incubation. This was done by evaluating the EPR spectral peak of FMX at t = 0 and t = 24 h and normalizing both FMX and FMX-L peaks to FMX alone. (**c**) Intracellular, chelatable iron content in U87 cells following a 24 h incubation with FMX or FMX-L. Error bars represent mean ± SEM with * *p* < 0.05 using a Welch’s *t*-test. (**d**) Representative phase contrast (40×) Prussian blue images for cellular iron content in U87 cells treated with FMX for 1 h and 24 h, or 24 h FMX-L. Black arrows indicate clusters of Prussian blue-positive cells.

A potential limitation of this approach is the extracellular nature of FMX [35]. Therefore, a proof-of-concept internalization model using lipofectamine was used to determine if T_2_* mapping can distinguish intracellular and extracellular FMX reduction by AscH^−^. To validate this model system, U87 cells were incubated with 20 µg mL^−1^ FMX ± lipofectamine (FMX-L) for 24 h. The initial observation made using this approach was that cell pellets following treatment with FMX-L had a reddish hue that would be indicative of high iron content (Figure 2a). Quantitatively, there was a significant decrease in FMX concentrations in the cell culture media, evaluated using EPR spectroscopy (Figure 2b) [14]. This indicates a shift of FMX from the extracellular to the intracellular space. The cell pellets also showed a significant, ≥3-fold, increase in iron concentrations (Figure 2c). This was further validated using Prussian blue staining where intracellular iron was markedly increased following FMX-L treatment (Figure 2d). Interestingly, an increase in Prussian blue positive cells was visible following a 1 h FMX incubation. This effect was not as pronounced by 24 h. This suggests an initial extracellular accumulation of FMX that dissipates over time. Lipofectamine appears to be a valuable tool for facilitating FMX internalization and intracellular retention.

### 3.3. FMX Internalization Enhances AscH^−^ Cytotoxicity

**Figure 3:**
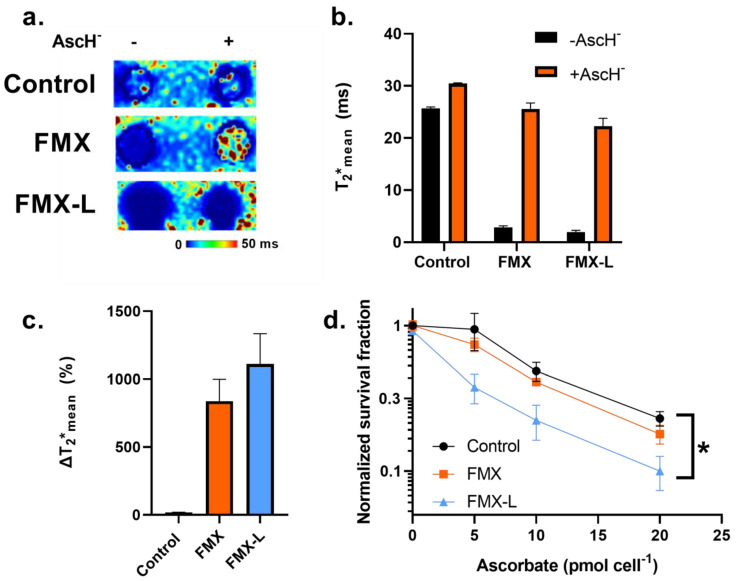
**FMX internalization enhances AscH^−^ cytotoxicity in glioblastoma cells.** (**a**) Representative T_2_* maps of U87 cell pellets treated with 20 pmol cell^−1^ AscH^−^ ± standard 1 h co-incubation with 20 µg mL^-1^ FMX or 24 h pre-treatment with 20 µg mL^−1^ FMX-L. (**b**) Mean T_2_* relaxation times in U87 cells treated with 20 pmol cell^−1^ AscH^−^ ± standard 1 h co-incubation with 20 µg mL^−1^ FMX or 24 h pre-treatment with 20 µg mL^−1^ FMX-L. (**c**) Changes in T_2_* relaxation time (% difference from untreated control) associated with 20 pmol cell^−1^ AscH^−^ treatment standard 1 h co-incubation with 20 µg mL^−1^ FMX or 24 h pre-treatment with 20 µg mL^−1^ FMX-L. (**d**) Clonogenic dose–response curves for U87 cells treated with increasing concentrations of AscH^−^ ± standard 1 h co-incubation with 20 µg mL^−1^ FMX or 24 h pre-treatment with 20 µg mL^−1^ FMX-L. Error bars represent mean ± SEM for three independent experiments with * *p* < 0.05 using a one-way ANOVA test with a post-hoc Tukey’s test.

This FMX internalization model system was used to evaluate if changes in T_2_* relaxation times reflect the internal reduction of FMX by AscH^−^. U87 cells were either co-incubated for 1 h with 20 µg mL^−1^ FMX ± 20 pmol cell^−1^ AscH^−^ or pre-treated for 24 h FMX-L to load the cells with FMX prior to their 1 h AscH^−^ treatment. Following treatment, cells were pelleted for T_2_* map generation. From this experiment, it has been observed that following a 1 h treatment with FMX or a 24 h treatment with FMX-L caused a noticeable signal loss, likely due to the ferromagnetic properties of FMX (Figure 3a). In both FMX and FMX-L treated cells, there was an observable susceptibility artifact surrounding the cell pellet that was much larger in the FMX-L cells, indicative of the significant increases in intracellular iron content that were previously described. AscH^−^-treated cells showed longer T_2_* relaxation properties; however, this was difficult to qualitatively visualize in the FMX-L treated cells due to the large signal loss. Quantitatively, AscH^−^ alone induced a 5 ms increase (control = 25.6 ms versus AscH^−^ = 30.4 ms) in T_2_ relaxation time, consistent with previous reports (Figure 3b) [27]. Both FMX and FMX-L cells caused a decrease in T_2_* relaxation time to 2.8 and 1.9 ms, respectively. This is consistent with the observed FMX deposition with both treatments. In both cases (FMX and FMX-L), AscH^−^ treated cells had significantly longer T_2_* relaxation times (25.6 and 22.3 ms, respectively). The T2* relaxation time change from baseline was significantly greater in those cells treated with FMX/FMX-L and AscH^−^ than AscH^−^ alone (Figure 3c). However, the internalization of FMX only partially increased the change in T_2_* by AscH^−^, suggesting that these doses of FMX for extracellular/intracellular differentiation were likely in the signal saturation range. Overall, these results further support the hypothesis that T_2_* relaxation time can detect the reduction of FMX by AscH^−^, but the high iron content of FMX may limit this effect.

Moreover, it has recently been reported that the combination of FMX and AscH^−^ exhibited enhanced cytotoxic effects in glioblastoma cells and significantly enhanced the standard of care therapy (radiation and temozolomide) in an in vivo animal model [19]. Thus, the therapeutic aspect of these imaging results was subsequently evaluated in glioblastoma cells. Based on the potential effects of FMX internalization on the ability of T_2_* to detect nanoparticle reduction, the effects on AscH^−^ toxicity were evaluated. Consistent with these imaging results, FMX-L significantly enhanced the dose-dependent AscH^−^ toxicity in U87 cells as FMX had a dose-enhancement ratio of 1.16 (*p* = 0.09) as compared to 1.54 for FMX-L (*p* < 0.05; Figure 3d). Thus, it appears that the internalization of FMX represents a novel strategy to enhance its utility in combination with AscH^−^; however, this may be a context-dependent effect that warrants further consideration.

## 4. Discussion

This study describes the ability of T_2_* mapping to detect the release of ferrous iron from FMX by AscH^−^. The primary utilization of FMX in the context of glioblastoma management is as an MR contrast agent [7,36,37]. FMX is also being investigated as a marker for glioblastoma progression [37]. Therefore, T_2_* may also be a valuable tool to identify regions of FMX accumulation. We demonstrate that FMX can decrease T_2_* relaxation times in vitro. This is consistent with previous data showing that FMX can decrease T_2_* relaxation times in humans 24 h following its administration likely owing to its 14–21 h intravascular half-life [7,38]. In this study, supraphysiological concentrations of AscH^−^ (10 mM), which are typically achieved via intravenous injection during glioblastoma therapy, were used [39,40]. Thus, this chemical combination more closely replicates an interaction that may be observed during glioblastoma therapy. Adding a reducing agent (AscH^−^) to FMX increases T_2_* relaxation times, which coincides with the release of Fe^2+^ from the nanoparticle core [19]. This is consistent with the iron oxidation state specificity of T_2_* mapping [29]. The oxidation state specificity of T_2_* mapping could be further validated in vitro in this study as AscH^−^ induces an increase in T_2_* relaxation while DFO causes a decrease. Importantly, this chemistry effect was able to be replicated in the context of AscH^−^ and FMX chemistry as the addition of AscH^−^ can prolong FMX relaxation times. This indicates that AscH^−^ can reduce the Fe^3+^ sites of FMX leading to an increase in the Fe^2+^:Fe^3+^ ratio, which can be detected with T_2_* mapping. These results are consistent with the increase in T_2_* associated with adding AscH^−^ to FMX in an orthotopic glioblastoma model [19]. Thus, the present study provides further insights into the ability of T_2_* mapping to detect the catalyzed release of Fe^2+^ from the Fe_3_O_4_ core by AscH^−^.

FMX and AscH^−^ chemistry was detected in both the extracellular and intracellular space with FMX internalization facilitated by lipofectamine. In this cell culture model, adding FMX caused a significant decrease in T_2_* regardless of its localization. The internalization did appear to shorten T2* relaxation times further, consistent with the significant increase in cellular iron content; however, detectable differences were challenging due to potential signal saturation. In both cases, FMX and FMX-L, adding AscH^−^ significantly increased T_2_* relaxation times. Following the internalization of FMX (FMX-L), the increase in T_2_* relaxation time induced by AscH^−^ was slightly greater but was ultimately limited by the potential signal saturation caused by FMX. Thus, it is important to note that due to the large size (≈30 nm) and high iron content of FMX, T_2_* relaxation appears to lose the ability to detect intracellular versus extracellular localization [19,41]. Therefore, the use of T_2_* may have an intrinsic technical limitation where the high iron concentrations of FMX limit the range of oxidation state specificity and impair the ability to evaluate FMX reduction by AscH^−^. This can be overcome by using ultrashort echo time (UTE)-T_2_* and may warrant further investigation [42].

Furthering the nanotheranostic potential of FMX and AscH^−^, the internalization of FMX significantly enhanced AscH^−^ toxicity. Thus, the internalization of FMX may significantly enhance the therapeutic utility in combination with AscH^−^ in GBM. Developmental efforts have been previously put forth to functionalize FMX and enhance tumor trafficking and internalization. For example, it has been shown that FMX functionalized with a Toll-like receptor 3 agonist enhanced melanoma tumor control [43]. Moreover, the trend towards a greater increase in T_2_* relaxation following internalization suggests that FMX reduction by AscH^−^ is driving the enhanced toxicity. These results are also consistent with previous literature that demonstrates increases in intracellular iron content enhance AscH^−^ toxicity [44]. This would support the hypothesis that cellular AscH^−^ uptake by sodium vitamin C transporters (SVCTs) mediate AscH^−^ toxicity in glioblastoma cells [45]. Therefore, it can be hypothesized that surface modifications of FMX to increase tumor trafficking and internalization can enhance the effectiveness of FMX and AscH^−^ in the management of GBM and warrant further investigation.

## 5. Conclusions

In summary, this study provides important insights into the utility of T_2_* mapping as a tool for assessing FMX and AscH^−^ chemistry in a biologically relevant model system. The large size of FMX can cause T_2_* signal saturation in GBM cells, limiting the ability to detect FMX internalization robustly. However, the oxidation state specificity of T_2_* mapping was partially retained. Moreover, the internalization of FMX significantly enhanced AscH^−^ toxicity in glioblastoma cells. Thus, FMX internalization strategies (e.g., surface modifications) may warrant further investigation as a therapeutic approach. These data help contextualize the nanotheranostic application of FMX and AscH^−^ therapy in glioblastoma to be considered in ongoing studies.

## Data Availability

Data is available upon request of the corresponding authors.
